# The J-shaped relationship between serum osmolality and all-cause mortality in critically ill patients with myocardial infarction: a retrospective cohort study

**DOI:** 10.3389/fendo.2025.1542403

**Published:** 2025-03-21

**Authors:** Long Gui, Heshan Cao, Min Zheng, Yu Pan, Chengdong Ning, Mingjin Cheng

**Affiliations:** ^1^ Department of Cardiovascular Surgery, Sun Yat-sen Memorial Hospital, Sun Yat-sen University, Guangzhou, Guangdong, China; ^2^ Department of Cardiothoracic Surgery, Lu ‘an Hospital Affiliated to Anhui Medical University, Lu ‘an, Anhui, China; ^3^ Department of Neurology, Sun Yat-sen Memorial Hospital, Sun Yat-sen University, Guangzhou, Guangdong, China

**Keywords:** serum osmolality, myocardial infarction, mortality, J-shaped association, MIMIC-IV

## Abstract

**Background:**

Serum osmolality (SOSM) is an indicator of hydration status and is associated with the prognosis of various cardiovascular diseases. This study investigated the association between SOSM and all-cause mortality in critically ill patients with myocardial infarction (MI).

**Methods:**

This retrospective cohort study utilized data from the Medical Information for Intensive Care-IV (MIMIC-IV) database, including critically ill patients with a primary diagnosis of MI. Patients were categorized into tertile groups based on the SOSM levels. Kaplan-Meier (K-M) survival analysis, multiple Cox regression models, restricted cubic spline (RCS) analysis, and threshold effect analysis were used to investigate the nonlinear relationship between all-cause mortality in critically ill patients with MI and SOSM.

**Results:**

A total of 5354 patients with MI were included. K-M survival analysis showed that the survival rate of the high SOSM group was significantly lower than that of the other groups, which was consistent with the results after IPTW correction (log-rank *P*<0.05). Multiple Cox regression confirmed that patients with high SOSM had significantly increased risk of death at 30-day [HR, 1.45 (95% CI 1.21–1.73) *P*<0.001], 180-day [HR, 1.32 (95% CI 1.15-1.53) *P*<0.001], and 365-day [HR, 1.31(95% CI1.15-1.49) *P*<0.001]. RCS analysis and threshold effect analysis showed a J-shaped relationship between SOSM and mortality risk, and the minimum threshold of SOSM was 286.28 mmol/L.

**Conclusions:**

This study revealed a J-shaped relationship between SOSM and all-cause mortality in critically ill MI patients, suggesting its potential as a prognostic marker for risk stratification.

## 1 Introduction

Myocardial infarction (MI) is a localized necrosis of the myocardium due to acute ischemia and hypoxia caused by interruption of blood flow in the coronary arteries, which has a high hospitalization and mortality rate, and imposes a serious burden on society. Despite the optimization of revascularization and pharmacological strategies in patients with MI, the incidence of major adverse cardiovascular events remains high, especially in critically ill patients (1). While conventional prognostic indicators for MI, such as cardiac biomarkers like troponin levels and imaging parameters like left ventricular ejection fraction (LVEF), have been widely studied (2, 3), identifying novel and effective risk stratification markers remains a key focus of current clinical practice.

Previous studies have shown that the prognosis of MI is not only affected by electrolyte levels (4–6), but also associated with changes in renal function and blood glucose (7–9). However, a single index is difficult to assess the prognosis of MI more accurately and is influenced by more external factors (10). Serum osmolality (SOSM), a composite measure of the body’s fluid and electrolyte balance, has emerged as a potential biomarker for prognosis of various disease (11–13). For instance, Rohla et al. (14) reported that high SOSM is associated with increased mortality in patients with acute coronary syndrome undergoing percutaneous coronary intervention (PCI).

In critically ill MI patients, cardiac function is often severely compromised, and they are more susceptible to conditions such as sodium retention and water-electrolyte disturbances (15–17). These pathophysiological changes can lead to cellular damage through dehydration or edema, further exacerbating myocardial stress and systemic inflammation. Given its role in reflecting underlying fluid-electrolyte homeostasis, SOSM may serve as a valuable indicator of disease severity. Notably, both excessively high and low SOSM levels may be detrimental, suggesting a potential U-shaped or J-shaped relationship with mortality.

Despite its potential clinical relevance, large-scale studies investigating the association between SOSM and mortality in critically ill MI patients remains insufficiently studied. This study aims to evaluate the prognostic value of SOSM in this high-risk population, providing insights that may improve early risk stratification and guide targeted clinical interventions.

## 2 Methods

### 2.1 Data sources and study population

We extracted anonymized medical record data from the MIMIC-IV v3.1 database for intensive care patients at Beth Israel Deaconess Medical Center (BIDMC) between 2008 and 2022, totaling 94,458 records. The database was developed jointly by the Massachusetts Institute of Technology (MIT), BIDMC, and Philips Healthcare (18, 19). With the approval of the Institutional Review Boards of BIDMC (protocol number 2001-P-001699/14) and MIT (protocol number 040300020206), we were authorized to collect and use the relevant data and were exempted from the step of obtaining informed consent. Study team member HS Cao has completed the necessary collaborative institutional training (certification number 63137030).

### 2.2 Inclusion and exclusion criteria

**Figure 1:**
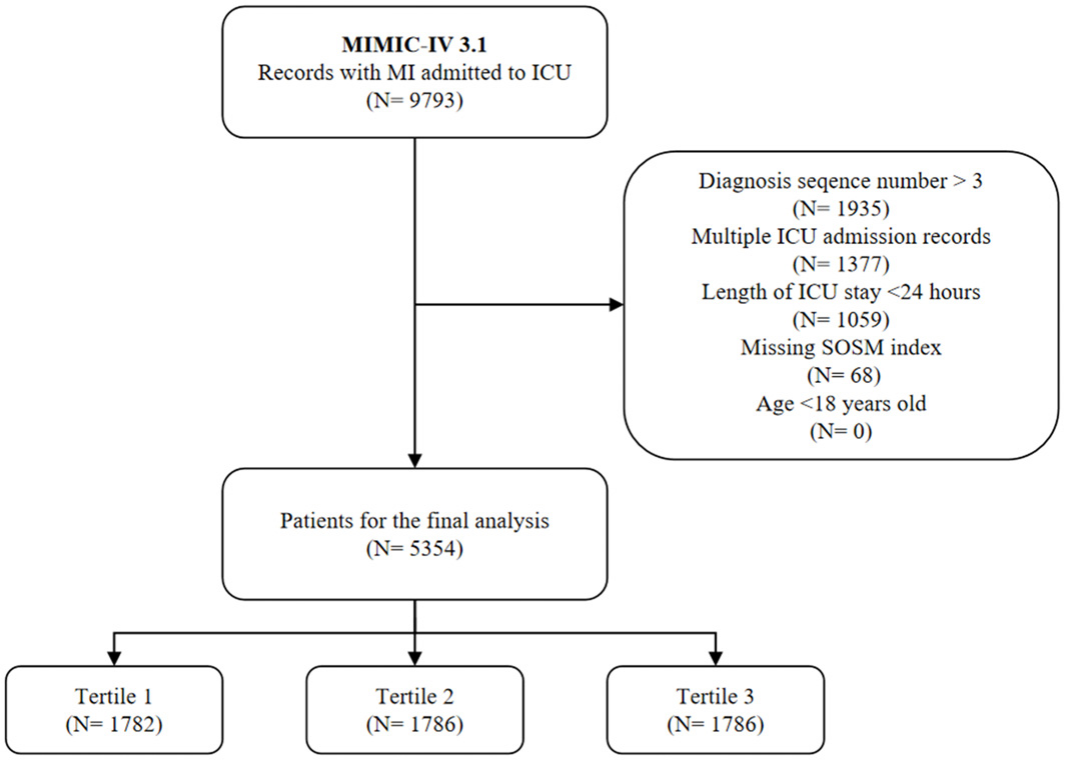
Study flow chart. MIMIC-IV, medical information mart for intensive care-IV; MI, myocardial infarction; ICU, intensive care unit; SOSM, serum osmolality.

We identified patients admitted to ICU with a primary diagnosis of MI based on International Classification of Diseases (ICD) 9/10 codes (**Supplementary Table S1**). Through the eligibility assessment, we excluded the population with the following criteria (1): age less than 18 years (2); the diagnostic sequence of MI was greater than 3 (3); non-first ICU admission and ICU stay time less than 24 hours (4); the relevant indicators for calculating SOSM, including sodium, potassium, blood glucose and blood urea nitrogen (Bun), were missing during the first 24 hours of ICU admission. The final 5354 patients were selected as the subjects of the study and divided according to the tertiles of SOSM (**Figure 1**).

### 2.3 Data collection and processing

The following variables were extracted from the MIMIC-IV database using Structured Query Language (SQL): (1) Demographics: age, gender, race, body mass index (BMI); (2) Vital signs: heart rate, mean blood pressure (MBP), respiratory rate, temperature; (3) Laboratory tests: sodium, potassium, glucose, hemoglobin, platelet, white blood cell (WBC), aniongap, creatinine, Bun, international normalized ratio (INR), partial thromboplastin time (PTT); (4) Comorbidities: hypertension, diabetes, diabetes with complication, congestive heart failure, cerebrovascular disease, peripheral vascular disease, chronic pulmonary disease, renal disease; (5) Clinical scores: sequential organ failure assessment (SOFA), acute physiology score III (APSIII), Oxford acute severity of illness score (OASIS); (6) Medication usage: vasoactive, antihypertensive, antilipemic, antiplatelet, insulin. SOSM was calculated as follows (20, 21):


SOSM=1.86×(sodium+potassium)+1.15×glucose+Bun+14

To reduce the possible variability caused by a single measurement, we calculated SOSM by averaging multiple measurements on the first day of ICU admission. Variables with >20% missing in the database were deleted. We used the mice package of R and a random forest approach to multiple imputation for variables with a missing rate of less than 20%. For details on missing covariates, see **Supplementary Figure S1** in the **Supplementary Material**. Data before and after imputation are shown in **Supplementary Table S2** in the **Supplementary Material**. To ensure the absence of multicollinearity between variables, we applied the variance inflation factor (VIF) test and excluded variables with VIF values greater than 5 (22) (**Supplementary Table S3**).

### 2.4 Outcomes

The primary outcome was short-term (30 days), medium-term (180 days), and long-term (365 days) all-cause mortality after ICU admission, with all-cause mortality defined as the proportion of patients who died from any cause during those specified time periods. In addition, we analyzed the following secondary outcomes: in-hospital mortality, incidence of severe acute kidney injury (AKI), length of ICU stay, and total length of hospital stay. Severe AKI followed the KDIGO criteria of grade II-III. AKI was defined as an increase in serum creatinine (SCr)inin mg/dL (ordLini µmol/L) within 48 hours, an increase in SCr to 1.5 times the baseline level within 7 days, and urine output ene.5 mL/kg/h within 6 hours. Detailed AKI staging definitions can be found in relevant clinical practice guidelines (23).

### 2.5 Statistical analysis

For continuous variables that follow a normal distribution, comparisons between groups were conducted using the t-test, with results reported as mean ± standard deviation. For continuous variables with non-normal distribution, the Mann-Whitney U test was applied, and results were presented as median (interquartile range, IQR). For categorical variables, comparisons between groups were made using the χ^2^ test or Fisher’s exact test, with results expressed as percentages. For multiple groups of continuous variables with normal distribution, analysis of variance (ANOVA) was used, while the Kruskal–Wallis H test was employed for variables with non-normal distribution. *Post hoc* pairwise comparisons for normal data were performed using the Tukey method, and the Dunn method was used for non-normal data, both with Bonferroni correction (a *P*-value < 0.05 was considered statistically significant). For comparisons of rates among multiple groups, the repeated χ² test was used, with the Bonferroni-corrected *P*-value serving as the reference standard (in this study, a *P*-value < 0.0167 among three groups was considered statistically significant).

To explore the association between SOSM and clinical outcomes, we first utilized Kaplan-Meier (K-M) survival curves, stratified by tertiles of SOSM, to analyze the incidence of primary outcome events across different SOSM levels. Differences between groups were compared using the log-rank test. The robustness of this relationship was verified by adjusting for confounding factors among the three groups using the inverse probability of treatment weighting (IPTW) method. Furthermore, we conducted in-depth exploration of the relationship between SOSM and the primary outcome using Cox regression analysis models. In this process, SOSM was analyzed as both a continuous and categorical variable across multiple models. Specifically, Model I did not adjust for any covariates; Model II adjusted for demographic characteristics, vital signs, and laboratory test results (hemoglobin, platelets, WBC, aniongap, creatinine, INR, PTT); and Model III further adjusted for comorbidities, clinical scores, and medication use in addition to the adjustments made in Model II. Additionally, we performed subgroup analyses stratified by age, gender, BMI, diabetes, renal disease, SOFA score, and insulin use to analyze potential interactions. To more closely examine the dose-effect relationship between SOSM and the risk of primary endpoint events, we employed restricted cubic spline (RCS) analysis and threshold analysis with SOSM as a continuous variable. Finally, for secondary outcomes, we performed univariate and multivariate logistic regression analyses and calculated the odds ratio (OR) to explore the relationship between SOSM and in-hospital mortality, and severe AKI incidence. Simultaneously, we further assessed the relationship between SOSM and the length of ICU stay and hospital stay through analysis of inter-group differences. All data analysis and visualization were completed using R language v4.2.3.

## 3 Results

### 3.1 Study population

A total of 5354 critically ill MI patients with an average age of 72.0 years (IQR: 63.0-81.0), 64.6% (3459) male and 63.6% (3405) white were included in this study. The 30-, 180-, and 365-day all-cause mortality rates were 16.1% (863), 24.0% (1286), and 28.8% (1543). According to the SOSM tertiles, the patients were divided into three groups: T1 group (245.10≤SOSM<286.32, n=1782), T2 group (286.32≤SOSM<292.2, n=1786) and T3 group (292.2≤SOSM<342.43, n=1786). Compared with the lower SOSM group, the patients in the higher SOSM group were older, had a lower proportion of males, and had higher heart rate, MBP, respiratory rate, temperature, sodium, glucose, aniongap, creatinine, Bun, INR, and PTT. In addition, patients in the higher SOSM group had more comorbidities (except for peripheral vascular disease) and worse clinical scores. However, the use of vasoactive drugs and antilipemic drugs was significantly lower in this group than in the lower SOSM group. The remaining baseline data are detailed in **Table 1**.

**Table 1:** Baseline characteristics according to SOSM tertile.

Variables	Total (n=5354)	T1 (n=1782)	T2 (n=1786)	T3 (n=1786)	*P-*value
Demographics					
Age, years	72.0 (63.0, 81.0)	71.0 (62.0, 79.0)	71.0 (61.0, 80.0)	74.0 (65.0, 83.0)	<0.001
Gender, Male, n (%)	3459 (64.6)	1178 (66.1)	1190 (66.6)	1091 (61.1)	<0.001
Race, White, n (%)	3405 (63.6)	1129 (63.4)	1133 (63.4)	1143 (64.0)	0.910
BMI	27.8 (24.3, 31.9)	27.7 (24.4, 31.3)	28.0 (24.6, 32.0)	27.8 (24.1, 32.4)	0.102
Vital signs					
Heart rate, bpm	81.0 (73.0, 90.8)	82.0 (73.0, 90.0)	80.0 (72.0, 89.0)	82.0 (72.0, 93.0)	0.020
MBP, mmHg	76.0 (71.0, 83.0)	75.0 (70.0, 81.0)	76.0 (71.0, 83.0)	77.0 (71.0, 84.0)	<0.001
Respiratory rate, bpm	19.0 (17.0, 21.0)	19.0 (17.0, 21.0)	19.0 (17.0, 21.0)	20.0 (17.0, 22.0)	<0.001
Temperature, °C	36.8 (36.6, 37.0)	36.7 (36.6, 37.0)	36.8 (36.6, 37.0)	36.8 (36.6, 37.0)	0.040
Laboratory tests					
Na^+^, mmol/L	138.0 (135.6, 140.0)	135.0 (133.0, 136.3)	138.3 (137.0, 139.3)	141.0 (139.0, 143.0)	<0.001
K^+^, mmol/L	4.3 (3.0, 4.6)	4.30 (4.0, 4.6)	4.30 (4.0, 4.6)	4.3 (3.9, 4.7)	0.425
Glucose, mmol/L	7.3 (6.2, 9.4)	6.6 (5.8, 7.9)	7.1 (6.2, 8.6)	8.9 (7.1, 12.1)	<0.001
Hemoglobin, g/dL	10.5 (9.1, 12.1)	10.3 (9.1, 11.8)	10.7 (9.4, 12.4)	10.4 (8.9, 12.2)	<0.001
Platelets, K/μL	189.0 (145.0, 242.0)	187.0 (143.0, 242.0)	190.0 (145.3, 238.0)	192.0 (146.0, 246.0)	0.410
WBC, K/μL	11.8 (9.1, 15.4)	11.9 (9.3, 15.4)	11.6 (9.1, 15.2)	12.0 (8.9, 15.7)	0.269
Aniongap, mmol/L	14.0 (12.0, 16.0)	13.0 (11.0, 16.0)	14.0 (11.0, 16.0)	15.0 (13.0, 18.0)	<0.001
Creatinine, mg/dL	1.1 (0.8, 1.8)	1.0 (0.8, 1.5)	1.0 (0.8, 1.5)	1.4 (1.0, 2.2)	<0.001
Bun, mmol/L	1.2 (0.8, 2.0)	1.0 (0.7, 1.6)	1.1 (0.8, 1.6)	1.7 (1.1, 2.8)	<0.001
INR	1.3 (1.1, 1.4)	1.3 (1.2, 1.4)	1.2 (1.1, 1.4)	1.3 (1.1, 1.5)	<0.001
PTT, s	35.4 (28.9, 57.6)	34.1 (28.5, 50.5)	34.6 (28.8, 54.8)	39.8 (29.3, 66.3)	<0.001
Comorbidities, n (%)					
Hypertension	2078 (38.8)	746 (41.9)	751 (42.1)	581 (32.5)	<0.001
Diabetes	2360 (44.1)	675 (37.9)	732 (41.0)	953 (53.4)	<0.001
Diabetes with complication	1084 (20.3)	303 (17.0)	297 (16.6)	484 (27.1)	<0.001
Congestive heart failure	2877 (53.7)	861 (48.3)	881 (49.3)	1135 (63.6)	<0.001
Cerebrovascular disease	756 (14.1)	207 (11.6)	221 (12.4)	328 (18.4)	<0.001
Peripheral vascular disease	858 (16.0)	278 (15.6)	279 (15.6)	301 (16.9)	0.505
Chronic pulmonary disease	1350 (25.2)	435 (24.4)	395 (22.1)	520 (29.1)	<0.001
Renal disease	1716 (32.1)	478 (26.8)	494 (27.7)	744 (41.7)	<0.001
Clinical scores					
SOFA	4.0 (2.0, 7.0)	4.0 (2.0, 6.0)	4.0 (2.0, 6.0)	5.0 (3.0, 8.0)	<0.001
APSIII	41.0 (31.0, 54.0)	38.0 (29.0, 52.0)	37.0 (28.0, 49.0)	48.0 (36.0, 61.0)	<0.001
OASIS	31.0 (26.0, 38.0)	31.0 (26.0, 37.0)	31.0 (25.0, 37.0)	33.0 (27.0, 40.0)	<0.001
Medications, n (%)					
Vasoactive	2462 (46.0)	849 (47.6)	836 (46.8)	777 (43.5)	0.032
Antihypertensive	4742 (88.6)	1587 (89.1)	1598 (89.5)	1557 (87.2)	0.071
Antilipemic	3857 (72.0)	1295 (72.7)	1313 (73.5)	1249 (69.9)	0.045
Antiplatelet	5047 (94.3)	1674 (94.0)	1684 (94.3)	1689 (94.6)	0.720
Insulin	2928 (54.7)	979 (55.0)	961 (53.8)	988 (55.3)	0.640
Outcomes					
30-day mortality, n (%)	863 (16.1)	218 (12.2)	188 (10.5)	457 (25.6)	<0.001
180-day mortality, n (%)	1286 (24.0)	348 (19.5)	310 (17.4)	628 (35.2)	<0.001
365-day mortality, n (%)	1543 (28.8)	419 (23.5)	385 (21.6)	739 (41.4)	<0.001
Hospital mortality, n (%)	655 (12.2)	179 (10.0)	130 (7.3)	346 (19.4)	<0.001
Severe AKI, n (%)	3138 (58.6)	961 (53.9)	978 (54.8)	1199 (67.1)	<0.001
ICU stay, days	2.40 (1.5, 4.2)	2.2 (1.4, 4.0)	2.2 (1.5, 4.0)	2.9 (1.8, 5.1)	<0.001
Hospital stay, days	8.1 (5.1, 12.8)	8.7 (5.8, 13.0)	7.8 (4.9, 12.0)	8.1 (4.9, 13.1)	<0.001

SOSM, serum osmolality; BMI, body mass index; MBP, mean blood pressure; Na+, sodium; K+, potassium; WBC, white blood cell; Bun, blood urea nitrogen; INR, international normalized ratio; PTT, partial thromboplastin time; SOFA, sequential organ failure assessment; APSIII, acute physiology score III; OASIS, Oxford acute severity of illness score; AKI, acute kidney injury; ICU, intensive care unit.

### 3.2 Primary outcomes

**Figure 2:**
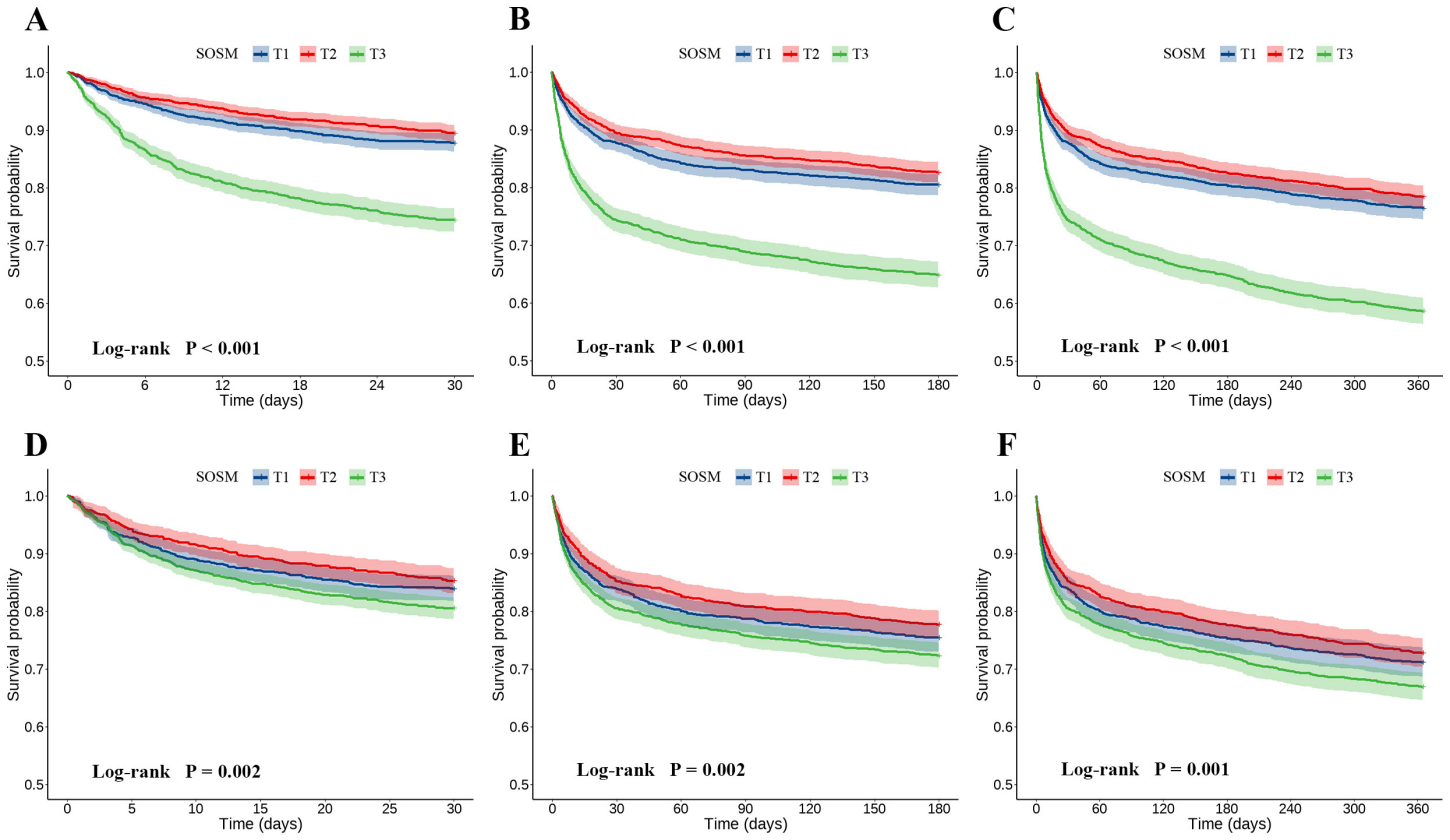
Kaplan-Meier (K-M) all-cause mortality survival analysis curve. **(A–C)** 30-, 180-, and 365-day K-M survival curves before IPTW; **(D–F)** 30-, 180-, and 365-day K-M survival curves after IPTW. SOSM, serum osmolality; IPTW, inverse probability of treatment weighting.

The 30-, 180-, and 365-day all-cause mortality rates in T3 group were 25.6%, 35.2% and 41.4%, respectively, which were significantly higher than those in T1 and T2 groups. This observation was further confirmed by the K-M survival analysis curves, which showed significant differences in the incidence of primary outcomes by SOSM tertiles during the short-, intermediate-, and long-term follow-up (log-rank *P*<0.05). To adjust for confounding factors, the IPTW method was used to ensure that covariates were balanced and comparable across groups (**Supplementary Table S4**, **Supplementary Figure S2**). The K-M survival analysis after IPTW adjustment showed that all-cause mortality at 30-, 180-, and 365-day remained statistically significant (log-rank *P* values were 0.002, 0.002, and 0.001, respectively) (**Figure 2**).

Multi-model Cox regression was used to analyze the association between SOSM and 30-day mortality. The results showed that SOSM was a significant risk factor in the unadjusted model [HR, 1.05 (95%CI 1.04-1.05), *P*<0.001], the partially model-adjusted model [HR, 1.02 (95%CI 1.01-1.03), *P*<0.001], and the fully adjusted model [HR, 1.01 (95%CI 1.01-1.02), *P<*0.001] for significant risk factors. When SOSM was the nominal variable (T2 as the reference), patients in higher tertiles of SOSM were significantly associated with a higher risk of 30-day death in three established Cox proportional risk models: unadjusted model [HR, 2.69 (95%CI 2.27-3.19), *P*<0.001], partially adjusted model [HR, 1.65 (95%CI 1.38-1.97), *P*<0.001] and fully adjusted models [HR, 1.45 (95%CI 1.21-1.73), *P*<0.001]. However, no statistical differences were found compared to the lowest tertile. Similar results were observed in the multi-model Cox proportional risk analysis for 180-day and 365-day mortality (**Table 2**). In multi-model analyses, the complexity of the model gradually increased as the corrected variables increased, and while HR was fine-tuned, the overall trend remained unchanged, with SOSM remaining a key predictor of mortality.

**Table 2:** Association of SOSM and the risk of all-cause mortality.

Categories		Model I			Model II			Model III		
		HR (95% CI)	*P*-value	*P* for trend	HR (95% CI)	*P*-value	*P* for trend	HR (95% CI)	*P*-value	*P* for trend
30-day mortality	SOSM (continuous)	1.05 (1.04-1.05)	<0.001		1.02 (1.01-1.03)	<0.001		1.01 (1.01-1.02)	<0.001	
	SOSM (tertiles)			<0.001			<0.001			<0.001
	T1	1.18 (0.97-1.44)	0.096		1.17 (0.96-1.42)	0.115		1.10 (0.91-1.35)	0.293	
	T2	Reference			Reference			Reference		
	T3	2.69 (2.27-3.19)	<0.001		1.65 (1.38-1.97)	<0.001		1.45 (1.21-1.73)	<0.001	
180-day mortality	SOSM (continuous)	1.04 (1.03-1.04)	<0.001		1.01 (1.01-1.02)	<0.001		1.01 (1.00-1.01)	0.007	
	SOSM (tertiles)			<0.001			<0.001			<0.001
	T1	1.15 (0.99-1.34)	0.076		1.14 (0.98-1.33)	0.087		1.09 (0.94-1.27)	0.382	
	T2	Reference			Reference			Reference		
	T3	2.33 (2.03-2.67)	<0.001		1.50 (1.30-1.72)	<0.001		1.32 (1.15-1.53)	<0.001	
365-day mortality	SOSM (continuous)	1.04 (1.03-1.04)	<0.001		1.01 (1.01-1.02)	<0.001		1.01 (1.00-1.01)	0.002	
	SOSM (tertiles)			<0.001			<0.001			<0.001
	T1	1.12 (0.97-1.28)	0.123		1.11 (0.97-1.28)	0.130		1.07 (0.93-1.23)	0.354	
	T2	Reference			Reference			Reference		
	T3	2.25 (1.99-2.55)	<0.001		1.46 (1.29-1.66)	<0.001		1.31 (1.15-1.49)	<0.001	<0.001

Model I: crude.

Model II: adjusted for demographics, vital signs and laboratory tests (hemoglobin, platelets, WBC, aniongap, creatinine, INR, PTT).

Model III: adjusted for demographics, vital signs, laboratory tests (hemoglobin, platelets, WBC, aniongap, creatinine, INR, PTT), clinical scores, comorbidities and medications.

SOSM, serum osmolality; HR, hazard ratio; CI, confidence interval; WBC, white blood cell; INR, international normalized ratio; PTT, partial thromboplastin time.

**Figure 3:**
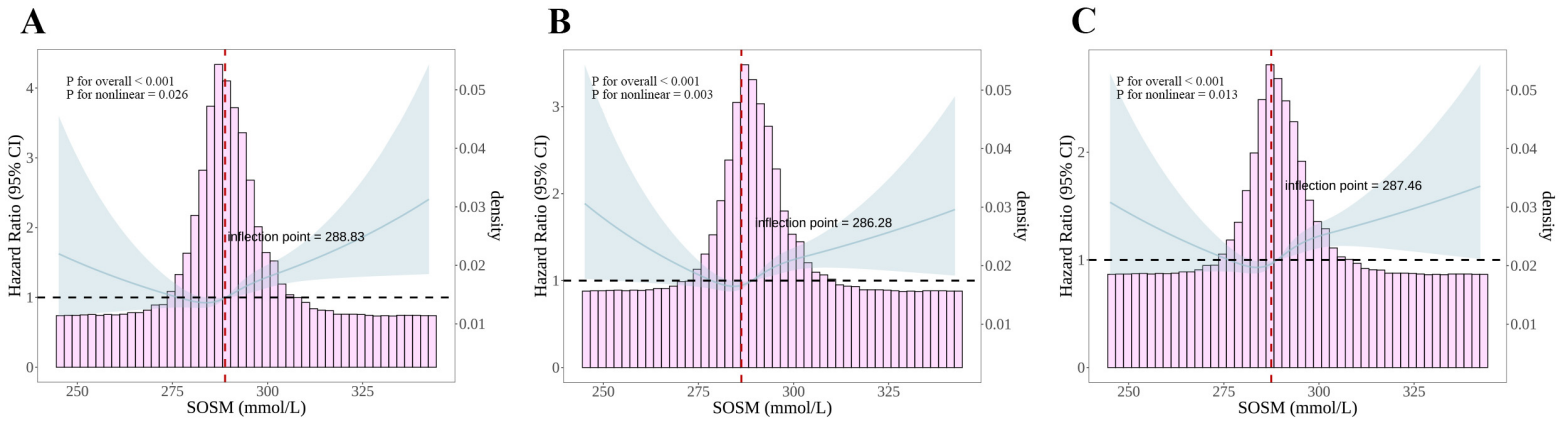
RCS plots of nonlinear analyses of SOSM and 30-, 180-, and 365-day all-cause mortality. **(A)** RCS plots between SOSM and 30-day mortality; **(B)** RCS plots between SOSM and 180-day mortality; **(C)** RCS plots between SOSM and 365-day mortality. Data were fitted by Cox proportional hazards regression models. Solid blue lines indicate HR and shaded shapes indicate 95% CI. RCS, restricted cubic spline; SOSM, serum osmolality; HR, hazard ratio; CI, confidence interval.

A J-shaped relationship between SOSM and the risk of short-, intermediate-, and long-term mortality in critically ill patients with MI was found by RCS analysis (*P* for nonlinear was 0.026, 0.003, and 0.013, respectively) (**Figure 3**). In a further threshold analysis, we further fitted the association between SOSM and mortality using a two-stage Cox proportional risk regression model. Notably, we identified thresholds for short-, intermediate-, and long-term mortality risk (**Table 3**). Specifically, when SOSM was greater than 288.83, we observed that a 1-unit increase in SOSM was associated with a 2.2% increase in the risk of short-term mortality [HR, 1.022 (95%CI 1.012-1.032), *P*<0.001]. A 1-unit increase in SOSM was associated with a 1.6% increase in the risk of intermediate-term mortality when SOSM was greater than 286.28 [HR, 1.016 (95%CI 1.009-1.024), *P*<0.001] as well as a 1-unit increase in SOSM was associated with a 1.6% increase in the risk of long-term mortality when SOSM was greater than 287.46 [HR, 1.016 (95%CI 1.008-1.023), *P*<0.001].

**Table 3:** Threshold effect analysis.

Outcome	30-day mortality		180-day mortality		365-day mortality	
	HR (95% CI)	*P-*value	HR (95% CI)	*P-*value	HR (95% CI)	*P-*value
Standard linear regression model	1.013 (1.006-1.020)	<0.001	1.008 (1.002-1.014)	0.007	1.009 (1.003-1.014)	0.002
Two-stage regression models						
Inflection point (K)	288.83		286.28		287.46	
< K	0.994 (0.979-1.010)	0.467	0.988 (0.975-1.002)	0.086	0.994 (0.982-1.006)	0.332
> K	1.022 (1.012-1.032)	<0.001	1.016 (1.009-1.024)	<0.001	1.016 (1.008-1.023)	<0.001
P for likelihood ratio test		0.011		0.003		0.011

HR, hazard ratio; CI, confidence interval.

### 3.3 Secondary outcomes

**Figure 4:**
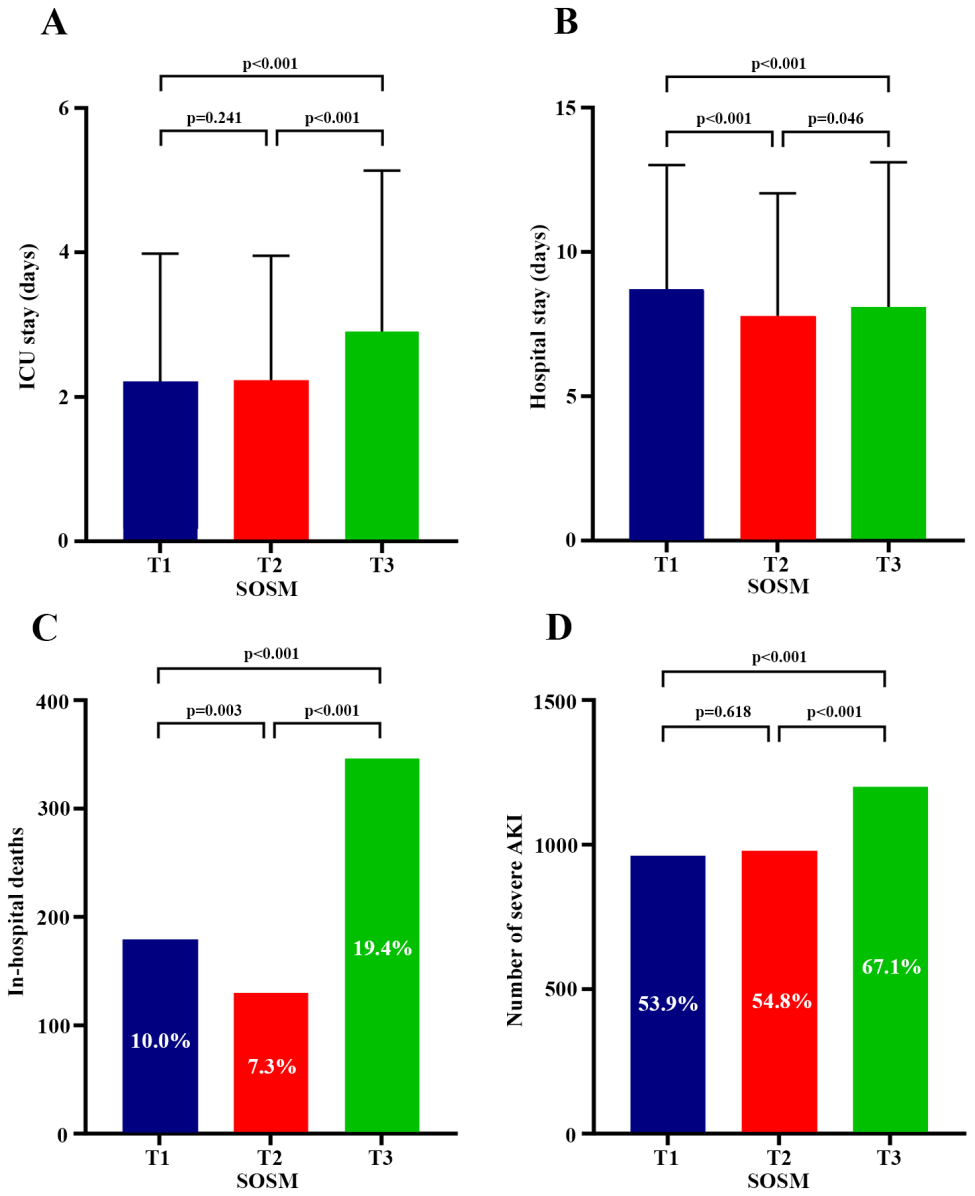
Plots of SOSM and relationship to secondary outcomes. **(A)** Comparison of ICU length of stay among the three groups; **(B)** Comparison of hospital length of stay among the three groups; **(C)** Comparison of in-hospital mortality among the three groups; **(D)** Comparison of the incidence of severe AKI among the three groups. *Post hoc* comparisons of continuous variables between the three groups were considered statistically significant at *P*<0.05. Comparisons of rates between the three groups were made using the repeated χ² test, with Bonferroni’s method corrected *P*<0.0167 considered statistically significant differences. SOSM, serum osmolality; ICU, intensive care unit; AKI, acute kidney injury.

There were statistically significant differences in in-hospital mortality, incidence of severe AKI, length of ICU stay, and length of hospital stay across SOSM tertiles (**Table 1**). Univariate and multivariate logistic regression analysis showed that both high and low SOSM were independent risk factors for increased in-hospital mortality compared with T2 group [T1 group: OR, 1.41 (95%CI 1.08-1.83), *P*=0.011; T3 group: OR, 1.60 (95%CI 1.25-2.04), *P*<0.001]. High SOSM was an independent risk factor for the increased incidence of severe AKI during hospitalization [OR, 1.22 (95%CI 1.04-1.42), *P*=0.012] (**Supplementary Tables S5, S6**), while low SOSM was not associated with the incidence of severe AKI. Relative to the T2 group, the high SOSM group had a longer length of both ICU and hospital stay, while the low SOSM group only showed a longer length of hospital stay, but not of ICU stay (**Figure 4**).

### 3.4 Subgroup analysis

**Figure 5:**
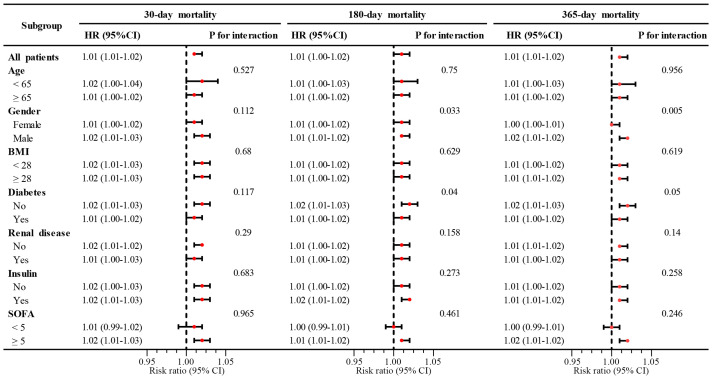
Subgroup analysis of SOSM with 30-, 180-, and 365-day mortality. SOSM, serum osmolality; HR, hazard ratio; CI, confidence interval; BMI, body mass index; SOFA, sequential organ failure assessment.

Subgroup analyses were performed according to age, sex, BMI, diabetes status, renal disease, SOFA score, and insulin use, with strict adjustment for other potential confounders. The results revealed that high SOSM significantly promoted the risk of mortality in critically ill patients with MI in all subgroups. In the interaction analysis, the *P* values for the interaction between gender and diabetes status in the assessment of all-cause mortality decreased gradually from 30 days to 365 days. Notably, all-cause mortality was more significant at 180 days and 365 days in male patients without concomitant diabetes, although short-term mortality did not demonstrate statistical differences between subgroups (**Figure 5**).

## 4 Discussion

This study is the first comprehensive investigation of the correlation between SOSM and all-cause mortality in critically ill patients with MI based on the MIMIC database. The results showed a significant J-shaped correlation between SOSM and the occurrence of short-, intermediate-, and long-term mortality in this patient population. Threshold effect analyses revealed inflection points for SOSM at 30-day mortality (288.83), 180-day mortality (286.28), and 365-day mortality (287.46). Even after adjusting for potential covariates, high SOSM levels were still associated with an increased risk of all-cause mortality and were significantly associated with higher rates of in-hospital mortality and severe AKI, longer length of both ICU and hospital stay.

Critically ill patients with MI are in a state of stress, and the body is able to activate the neuroendocrine system and trigger a redistribution of blood flow (24). On the one hand, the activation of renin-angiotensin-aldosterone system leads to increased levels of vasopressin, brain natriuretic peptide, catecholamines and other hormones, which may be accompanied by renal dysfunction (25). On the other hand, the rapid reduction of coronary blood flow leads to a large amount of lactate production, which affects the function of sodium-potassium transporters, and then alters hormone levels and sodium-potassium balance, eventually leading to abnormal SOSM and poor prognosis (26). However, these pathophysiological changes make it difficult to evaluate the prognosis of critically ill MI patients with a single indicator. SOSM is a clinical parameter that is simple to calculate and easy to obtain. It integrates the comprehensive information of electrolytes, blood glucose and Bun, and has been confirmed to have good clinical predictive value (27, 28). In a study on the relationship between SOSM and the prognosis of ACS patients, Rohla et al. (14) found that after adjusting for confounding factors, high SOSM was associated with the risk of in-hospital death in the study population [HR, 2.75 (95%CI 1.35-5.61), *P*=0.005], 30-day mortality [HR, 2.53 (95%CI 1.23-5.21), *P*=0.012], and 1-year mortality [HR, 1.73 (95%CI 1.02-2.91), *P*=0.04]. When critically ill patients were excluded, the association disappeared, and the authors did not further analyze the heterogeneity between ordinary and severe patients. Our study was the first to focus on critically ill patients with MI, and K-M analysis showed that there were differences in survival curves among the three groups, with patients in the high SOSM group having worse survival at 30-, 180-, and 365-day of follow-up, which remained robust after IPTW method was used to balance the confounding factors between groups. In addition, in the multivariate Cox regression analysis, we gradually constructed a nested model to confirm that high SOSM could increase the risk of short-, intermediate-, and long-term all-cause mortality, which was consistent with the analysis results of Shen et al. (29), who studied the heart disease group in critically ill patients.

The specific mechanisms underlying the effect of SOSM on mortality have not been fully defined (30). Numerous studies have shown that high levels of SOSM can increase the concentration of pro-inflammatory cytokines, including interleukin-8 (IL-8), IL-6, IL-1β, and tumor necrosis factor-α. At the same time, high osmolality of extracellular fluid affects neutrophil activity and leukotriene production (31, 32), suggesting that changes in SOSM may regulate the activity of the proinflammatory immune system. In addition, the hypertonic state of serum also drives the transfer of water to the extracellular space, leading to cell shrinkage. These changes in cell morphology have the potential to damage key structures inside the cell, such as the nucleus and mitochondria, which could eventually trigger cell death (33, 34). High SOSM also reflects greater fluid deficit and maladaptation, especially when combined with metabolic acidosis and its vascular paralysis (35). When the body is in low SOSM, the cell volume expands and may even rupture, the cell contents leak, and eventually cause organ dysfunction. In a cohort study of 20,160 patients (36), it was shown that early high and low SOSM were independently associated with increased risk of severe AKI. Liang et al. (13) also found a U-shaped relationship between serum osmolality and 28- and 90-day mortality risk in patients with sepsis, and lower or higher SOSM levels were associated with increased risk of death. However, low SOSM was not associated with increased mortality and readmission rates for heart failure as reported by Arévalo-Lorido et al. (37).

We found a J-shaped relationship between SOSM and all-cause mortality in the fitting RCS curve (*P* for nonlinear <0.05). Further threshold analysis helped us to find a specific inflection point of SOSM. In the short term observation, all-cause mortality in patients with severe MI gradually increased when SOSM exceeded 288.83 mmol/L. This finding suggests that clinicians should take aggressive interventions when SOSM exceeds this threshold. These interventions include closer monitoring, prompt medication or symptomatic treatment to reduce a patient’s risk of death and improve their prognosis. Although there was no statistically significant difference between low SOSM and all-cause mortality compared to the T2 group, our analysis showed that either too high or too low SOSM may increase in-hospital mortality and prolong hospital stay, which is consistent with the findings of some other investigators. This association may be influenced by a number of factors, including differences in participants included in the study, diversity of pathological states, and sample size. At present, the relationship between low SOSM and increased risk of death is unclear and needs to be validated and clarified with more research (36, 38). High SOSM may lead to blood concentration and increased viscosity, affect renal blood perfusion, and reduce glomerular filtration rate, which can lead to AKI and prolonged ICU stay. In addition, high SOSM may directly damage renal tubule cells, lead to cell dysfunction or death, and then affect renal reabsorption and secretion functions, and promote the occurrence of AKI (29).

In subgroup analyses, high SOSM was a risk factor for enhanced mortality regardless of gender and concomitant status of diabetes, and although there was no difference in 30-day mortality among groups, it is noteworthy that men without diabetes showed a higher mortality rate at intermediate- to long-term follow-up. There are many external factors causing hyperosmolality, and blood glucose, as one of the parameters used to calculate SOSM, is more significant in people with or without diabetes. Kosiborod et al. (39) confirmed that hyperosmolality caused by high blood glucose has harmful effects on the long-term survival of MI patients, especially in non-diabetic patients, in a retrospective study of 141,680 elderly patients. The current study did not find that gender differences directly affect changes in SOSM. However, sex may influence an individual’s physiological response to hyperosmotic pressure by influencing hormone levels and metabolic differences.

The limitations of this study are as follows: First, it is a large, single-center retrospective study, and although we adjusted for potential confounders to ensure the stability of the results, we could not establish causation. The analysis and conclusions of this study are based on known variables in the database and do not consider the potential impact of unmeasured or unknown variables. Second, while baseline SOSM provides valuable initial insights, a dynamic assessment of SOSM may provide more comprehensive information on the relationship between this measure and poor outcomes in patients. The dynamic change of SOSM, its molecular mechanism and its combined prediction value with other indexes need to be further explored. Finally, it is worth exploring to conduct multicenter prospective studies or randomized controlled trials for further verification in the future.

## 5 Conclusion

There was a J-shaped relationship between SOSM and all-cause mortality in critically ill patients with MI. High SOSM increased the risk of short-, intermediate-, and long-term mortality, prolonged the length of both ICU and hospital stay, and was also independently associated with a higher incidence of severe AKI and hospital mortality. These findings help to identify high-risk individuals early, optimize clinical management strategies, and improve patient outcomes.

## Data Availability

Publicly available datasets were analyzed in this study. This data can be found here: https://mimic.mit.edu/.
