# Supplementary material for: The J-shaped relationship between serum osmolality and all-cause mortality in critically ill patients with myocardial infarction: a retrospective cohort study
Source: Front Endocrinol (Lausanne). 2025 Mar 21;16:1542403. doi: 10.3389/fendo.2025.1542403 (PMC11968384; doi:10.3389/fendo.2025.1542403)
Supplement: Supplementary file 1 [file DataSheet1.docx]

**The J-shaped relationship between serum osmolality and all-cause mortality in critically ill patients with myocardial infarction: a retrospective cohort study**

**Supplementary material**

**Table S1.** The international classification of diseases (ICD) codes used in the study.

**Table S2.** Comparisons before and after imputation of missing data.

**Table S3.** Covariance analysis between variables.

**Table S4.** The details of covariates before / after adjustment by IPTW.

**Table S5.** Univariate and multivariate logistic regression analysis of factors influencing hospital mortality.

**Table S6.** Univariate and multivariate logistic regression analysis of factors influencing AKI.

**Figure S1.** Percentage of missing data in the variables included in this study.

**Figure S2.** Love plot of covariates before / after adjustment by IPTW among three groups.

| **Disease** | **Version** | **ICD code** |
| --- | --- | --- |
| Myocardial infarction | ICD-9 | 41000, 41001, 41002, 41010, 41011, 41012, 41020, 41021, 41022, 41030, 41031, 41032, 41040, 41041, 41042, 41050, 41051, 41052, 41060, 41061, 41062, 41070, 41071, 41072, 41080, 41081, 41082, 41090, 41091, 41092 |
|  | ICD-10 | I21, I210, I2101, I2102, I2109, I211, I2111, I2119, I212, I2121, I2129, I213, I214, I219, I21A, I21A1, I21A9, I22, I220, I221, I222, I228, I229 |

**Table S1. The international classification of diseases (ICD) codes used in the study.**

| **Variables** | **Before imputation** | |  | **After imputation** | | ***P*-value** |
| --- | --- | --- | --- | --- | --- | --- |
| BMI, kg/m^2^ | n = 4733 | 27.9 [24.4, 31.9] |  | n = 5354 | 27.8 [24.3, 31.9] | 0.673 |
| Heart rate, bpm | n = 5349 | 81.0 [73.0, 91.0] |  | n = 5354 | 81.0 [73.0, 90.8] | 0.987 |
| MBP, mmHg | n = 5349 | 76.0 [71.0, 83.0] |  | n = 5354 | 76.0 [71.0, 83.0] | 0.955 |
| Respiratory rate, bpm | n = 5346 | 19.0 [17.0, 21.0] |  | n = 5354 | 19.0 [17.0, 21.0] | 0.979 |
| Temperature, ℃ | n = 5122 | 36.8 [36.6, 37.0] |  | n = 5354 | 36.8 [36.6, 37.0] | 0.984 |
| Hemoglobin, g/dL | n = 5333 | 10.5 [9.1, 12.1] |  | n = 5354 | 10.5 [9.1, 12.1] | 0.946 |
| Platelets, K/μL | n = 5335 | 189.0 [145.0, 242.0] |  | n = 5354 | 189.0 [145.0, 242.0] | 0.958 |
| WBC, K/μL | n = 5332 | 11.8 [9.1, 15.4] |  | n = 5354 | 11.8 [9.1, 15.4] | 0.950 |
| Aniongap, mmol/L | n = 5351 | 14.0 [12.0, 16.0] |  | n = 5354 | 14.0 [12.0, 16.0] | 0.989 |
| INR | n = 4968 | 1.3 [1.1, 1.4] |  | n = 5354 | 1.3 [1.1, 1.4] | 0.290 |
| PTT, s | n = 4992 | 35.3 [28.8, 57.4] |  | n = 5354 | 35.4 [28.9, 57.6] | 0.876 |
| SOFA | n = 5349 | 4.0 [2.0, 7.0] |  | n = 5354 | 4.0 [2.0, 7.0] | 0.990 |

**Table S2. Comparisons before and after imputation of missing data.**

Abbreviations: BMI, body mass index; MBP, mean blood pressure; WBC, white blood cell; INR, international normalized ratio; PTT, partial thromboplastin time; SOFA, sequential organ failure assessment.

**Table S3**. **Covariance analysis between variables.**

| **Variable** | **VIF** | **Variable** | **VIF** |
| --- | --- | --- | --- |
| SOSM | 1.151 | Hypertension | 1.563 |
| Age | 1.432 | Diabetes | 1.715 |
| Gender | 1.141 | Diabetes with complication | 1.796 |
| Race | 1.055 | Congestive heart failure | 1.330 |
| BMI | 1.124 | Cerebrovascular disease | 1.047 |
| Heart rate | 1.331 | Peripheral vascular disease | 1.055 |
| MBP | 1.332 | Chronic pulmonary disease | 1.057 |
| Respiratory rate | 1.339 | Renal disease | 2.079 |
| Temperature | 1.113 | SOFA | 3.254 |
| Hemoglobin | 1.440 | APSIII | 2.674 |
| Platelets | 1.233 | OASIS | 2.200 |
| WBC | 1.127 | Vasoactive drugs | 1.616 |
| Aniongap | 1.765 | Antihypertensive | 1.088 |
| Creatinine | 1.893 | Antilipemic | 1.164 |
| INR | 1.105 | Antiplatelet drugs | 1.062 |
| PTT | 1.105 | Insulin | 1.562 |

Abbreviations: VIF, variance inflation factor; SOSM, serum osmolality; BMI, body mass index; MBP, mean blood pressure; WBC, white blood cell; INR, international normalized ratio; PTT, partial thromboplastin time; SOFA, sequential organ failure assessment; APSIII, acute physiology score III; OASIS, Oxford acute severity of illness score.

**Table S4. The details of covariates before / after adjustment by IPTW.**

| **Covariate** | **Before IPTW** | | | | | **After IPTW** | | | | |
| --- | --- | --- | --- | --- | --- | --- | --- | --- | --- | --- |
|  | **T1 (n=1782)** | **T2 (n=1786)** | **T3 (n=1786)** | ***P-*value** | **SMD** | **T1 (n=1800.7 )** | **T2 (n=1796.2)** | **T3 (n=1753.3)** | ***P-*value** | **SMD** |
| Age (median [IQR]) | 71.0 [62.0,79.0] | 71.0 [61.0,80.0] | 74.0 [65.0,83.0] | <0.001 | 0.181 | 72.0 [63.0,80.0] | 72.0 [63.0,81.0] | 72.0 [62.0,81.0] | 0.933 | 0.003 |
| Gender,Male (%) | 1178 (66.1) | 1190 (66.6) | 1091 (61.1) | 0.001 | 0.077 | 1155.8 (64.2) | 1152.5 (64.2) | 1110.4 (63.3) | 0.867 | 0.012 |
| Race,White (%) | 1129 (63.4) | 1133 (63.4) | 1143 (64.0) | 0.91 | 0.009 | 1155.6 (64.2) | 1140.8 (63.5) | 1118.6 (63.8) | 0.934 | 0.009 |
| BMI (median [IQR]) | 27.7 [24.4,31.3] | 28.0 [24.6,32.0] | 27.8 [24.1,32.4] | 0.102 | 0.044 | 27.9 [24.5,31.7] | 27.9 [24.4,32.0] | 27.7 [24.1,32.1] | 0.624 | 0.011 |
| Heart rate (median [IQR]) | 82.0 [73.0,90.0] | 80.0 [72.0,89.0] | 82.0 [72.0,93.0] | 0.002 | 0.094 | 81.0 [73.0,90.0] | 81.0 [73.0,91.0] | 81.0 [72.0,91.0] | 0.783 | 0.004 |
| MBP (median [IQR]) | 75.0 [70.0,81.0] | 76.0 [71.0,83.0] | 77.0 [71.0,84.0] | <0.001 | 0.139 | 76.0 [71.0,82.0] | 76.0 [71.0,83.0] | 76.0 [70.0,83.0] | 0.956 | 0.007 |
| Respiratory rate (median [IQR]) | 19.0 [17.0,21.0] | 19.0 [17.0,21.0] | 20.0 [17.0,22.0] | <0.001 | 0.204 | 19.0 [17.0,21.0] | 19.0 [17.0,21.0] | 19.0 [17.0,22.0] | 0.874 | 0.009 |
| Temperature (median [IQR]) | 36.7 [36.6,37.0] | 36.8 [36.6,37.0] | 36.8 [36.6,37.0] | 0.004 | 0.025 | 36.8 [36.6,37.0] | 36.8 [36.6,37.0] | 36.8 [36.6,37.0] | 0.086 | 0.004 |
| Hemoglobin (median [IQR]) | 10.3 [9.1,11.8] | 10.7 [9.4,12.4] | 10.4 [8.9,12.2] | <0.001 | 0.138 | 10.4 [9.1,12.0] | 10.4 [9.2,12.0] | 10.4 [9.0,12.2] | 0.862 | 0.013 |
| Platelets (median [IQR]) | 187.0 [143.0,242.0] | 190.0 [145.3,238.0] | 192.0 [146.0,246.0] | 0.41 | 0.022 | 189.0 [144.6,243.0] | 190.0 [144.0,242.0] | 191.0 [146.0,244.0] | 0.794 | 0.009 |
| WBC (median [IQR]) | 11.9 [9.3,15.4] | 11.6 [9.1,15.2] | 12.0 [8.9,15.7] | 0.269 | 0.027 | 11.8 [9.2,15.5] | 11.7 [9.1,15.4] | 11.90 [8.9,15.4] | 0.751 | 0.016 |
| Aniongap (median [IQR]) | 13.0 [11.0,16.0] | 14.0 [11.0,16.0] | 15.0 [13.0,18.0] | <0.001 | 0.405 | 14.0 [12.0,17.0] | 14.0 [12.0,16.0] | 14.0 [12.0,16.0] | 0.406 | 0.032 |
| Creatinine (median [IQR]) | 1.0 [0.8,1.5] | 1.0 [0.8,1.5] | 1.4 [1.0,2.2] | <0.001 | 0.184 | 1.1 [0.8,1.8] | 1.1 [0.9,1.8] | 1.2 [0.9,1.9] | 0.002 | 0.011 |
| INR (median [IQR]) | 1.3 [1.2,1.4] | 1.2 [1.1,1.4] | 1.3 [1.1,1.5] | <0.001 | 0.111 | 1.3 [1.2,1.4] | 1.3 [1.1,1.4] | 1.3 [1.1,1.4] | 0.011 | 0.008 |
| PTT (median [IQR]) | 34.1 [28.5,50.5] | 34.6 [28.8,54.8] | 39.8 [29.3,66.3] | <0.001 | 0.191 | 36.2 [29.0,57.6] | 35.5 [29.1,56.9] | 35.8 [28.9,59.3] | 0.946 | 0.016 |
| Hypertension=Yes (%) | 746 (41.9) | 751 (42.1) | 581 (32.5) | <0.001 | 0.132 | 685.2 (38.1) | 685.6 (38.2) | 643.3 (36.7) | 0.656 | 0.02 |
| Diabetes with complication=Yes (%) | 303 (17.0) | 297 (16.6) | 484 (27.1) | <0.001 | 0.17 | 792.9 (44.0) | 795.1 (44.3) | 763.3 (43.5) | 0.922 | 0.01 |
| Diabetes=Yes (%) | 675 (37.9) | 732 (41.0) | 953 (53.4) | <0.001 | 0.209 | 369.5 (20.5) | 365.4 (20.3) | 358.0 (20.4) | 0.993 | 0.003 |
| Congestive heart failure=Yes (%) | 861 (48.3) | 881 (49.3) | 1135 (63.6) | <0.001 | 0.207 | 991.8 (55.1) | 970.3 (54.0) | 969.2 (55.3) | 0.767 | 0.017 |
| Cerebrovascular disease=Yes (%) | 207 (11.6) | 221 (12.4) | 328 (18.4) | <0.001 | 0.127 | 255.3 (14.2) | 253.8 (14.1) | 252.1 (14.4) | 0.979 | 0.005 |
| Peripheral vascular disease=Yes (%) | 278 (15.6) | 279 (15.6) | 301 (16.9) | 0.505 | 0.023 | 294.3 (16.3) | 286.8 (16.0) | 285.3 (16.3) | 0.958 | 0.007 |
| Chronic pulmonary disease=Yes (%) | 435 (24.4) | 395 (22.1) | 520 (29.1) | <0.001 | 0.107 | 469.9 (26.1) | 453.3 (25.2) | 453.9 (25.9) | 0.86 | 0.013 |
| Renal disease=Yes (%) | 478 (26.8) | 494 (27.7) | 744 (41.7) | <0.001 | 0.211 | 594.7 (33.0) | 586.3 (32.6) | 593.1 (33.8) | 0.797 | 0.017 |
| SOFA (median [IQR]) | 4.0 [2.0,6.0] | 4.0 [2.0,6.0] | 5.0 [3.0,8.0] | <0.001 | 0.187 | 4.0 [2.0,7.0] | 4.0 [2.0,7.0] | 4.0 [2.0,7.0] | 0.975 | 0.004 |
| APSIII (median [IQR]) | 38.0 [29.0,52.0] | 37.0 [28.0,49.0] | 48.0 [36.0,61.0] | <0.001 | 0.344 | 41.0 [30.0,56.0] | 41.0 [30.0,55.0] | 43.0 [33.0,54.0] | 0.081 | 0.014 |
| OASIS (median [IQR]) | 31.0 [26.0,37.0] | 31.0 [25.0,37.0] | 33.0 [27.0,40.0] | <0.001 | 0.209 | 32.0 [26.0,38.0] | 32.0 [26.0,38.0] | 32.0 [26.0,38.0] | 0.984 | 0.006 |
| Vasoactive=Yes (%) | 849 (47.7) | 836 (46.8) | 777 (43.5) | 0.032 | 0.055 | 825.6 (45.9) | 821.7 (45.8) | 813.5 (46.4) | 0.932 | 0.009 |
| Antihypertensive=Yes (%) | 1587 (89.1) | 1598 (89.5) | 1557 (87.2) | 0.071 | 0.048 | 1586.0 (88.1) | 1582.8 (88.1) | 1536.8 (87.7) | 0.923 | 0.01 |
| Antilipemic=Yes (%) | 1295 (72.7) | 1313 (73.5) | 1249 (69.9) | 0.045 | 0.053 | 1275.9 (70.9) | 1293.5 (72.0) | 1236.7 (70.5) | 0.664 | 0.022 |
| Antiplatelet=Yes (%) | 1674 (93.9) | 1684 (94.3) | 1689 (94.6) | 0.72 | 0.018 | 1692.8 (94.0) | 1696.2 (94.4) | 1653.9 (94.3) | 0.878 | 0.012 |
| Insulin=Yes (%) | 979 (54.9) | 961 (53.8) | 988 (55.3) | 0.64 | 0.02 | 955.4 (53.1) | 983.7 (54.8) | 897.5 (51.2) | 0.158 | 0.048 |

Abbreviations: IPTW, inverse probability of treatment weighting; SMD, standardized mean difference; BMI, body mass index; MBP, mean blood pressure; WBC, white blood cell; INR, international normalized ratio; PTT, partial thromboplastin time; SOFA, sequential organ failure assessment; APSIII, acute physiology score III; OASIS, Oxford acute severity of illness score.

**Table S5. Univariate and multivariate logistic regression analysis of factors influencing hospital mortality.**

| **Variables** | **Univariate analysis** | |  | **Multivariate analysis** | |
| --- | --- | --- | --- | --- | --- |
|  | **OR (95%CI)** | ***P-*value** |  | **OR (95%CI)** | ***P-*value** |
| SOSM |  |  |  |  |  |
| T1 | 1.42 (1.12-1.80) | 0.003 |  | 1.41 (1.08-1.83) | 0.011 |
| T2 | Reference |  |  | Reference |  |
| T3 | 3.06 (2.47-3.79) | <0.001 |  | 1.60 (1.25-2.04) | <0.001 |
| Age | 1.03 (1.02-1.04) | <0.001 |  | 1.04 (1.03-1.05) | <0.001 |
| Gender, Male | 0.83 (0.71-0.99) | 0.035 |  |  |  |
| Race, White | 1.24 (1.05-1.47) | 0.01 |  | 1.30 (1.07-1.59) | 0.009 |
| BMI | 0.99 (0.98-1.01) | 0.428 |  |  |  |
| Heart Rate | 1.02 (1.02-1.03) | <0.001 |  | 1.01 (1.01-1.02) | 0.012 |
| MBP | 0.97 (0.96-0.98) | <0.001 |  |  |  |
| Respiratory Rate | 1.18 (1.15-1.20) | <0.001 |  | 1.08 (1.05-1.11) | <0.001 |
| Temperature | 0.62 (0.54-0.72) | <0.001 |  | 0.73 (0.63-0.85) | <0.001 |
| Hemoglobin | 0.93 (0.89-0.97) | <0.001 |  |  |  |
| Platelets | 1.00 (1.00-1.00) | 0.432 |  |  |  |
| WBC | 1.03 (1.02-1.04) | <0.001 |  |  |  |
| Aniongap | 1.22 (1.20-1.25) | <0.001 |  | 1.11 (1.08-1.14) | <0.001 |
| Creatinine | 1.19 (1.14-1.24) | <0.001 |  |  |  |
| INR | 1.63 (1.47-1.81) | <0.001 |  |  |  |
| PTT | 1.01 (1.01-1.02) | <0.001 |  | 1.01 (1.01-1.01) | <0.001 |
| Hypertension | 0.52 (0.43-0.63) | <0.001 |  | 0.60 (0.47-0.77) | <0.001 |
| Diabetes | 1.05 (0.89-1.24) | 0.541 |  |  |  |
| Diabetes with complication | 1.08 (0.89-1.32) | 0.443 |  |  |  |
| Congestive heart failure | 1.88 (1.59-2.24) | <0.001 |  |  |  |
| Cerebrovascular disease | 1.54 (1.24-1.90) | <0.001 |  | 1.53 (1.19-1.95) | <0.001 |
| Peripheral vascular disease | 1.43 (1.16-1.75) | <0.001 |  |  |  |
| Chronic pulmonary disease | 1.55 (1.30-1.84) | <0.001 |  | 1.39 (1.14-1.71) | 0.001 |
| Renal disease | 1.59 (1.34-1.88) | <0.001 |  | 0.59 (0.47-0.75) | <0.001 |
| SOFA | 1.28 (1.25-1.32) | <0.001 |  | 1.07 (1.03-1.12) | 0.002 |
| APSIII | 1.05 (1.04-1.05) | <0.001 |  | 1.02 (1.02-1.03) | <0.001 |
| OASIS | 1.10 (1.09-1.11) | <0.001 |  |  |  |
| Vasoactive | 2.57 (2.16-3.05) | <0.001 |  | 1.74 (1.38-2.19) | <0.001 |
| Antihypertensive | 0.49 (0.39-0.61) | <0.001 |  | 0.72 (0.55-0.94) | 0.015 |
| Antilipemic | 0.54 (0.46-0.64) | <0.001 |  | 0.69 (0.57-0.85) | <0.001 |
| Antiplatelet | 1.13 (0.78-1.62) | 0.524 |  |  |  |
| Insulin | 0.90 (0.76-1.06) | 0.203 |  |  |  |

Abbreviations: OR, odds ratio; CI: confidence interval; SOSM, serum osmolality; BMI, body mass index; MBP, mean blood pressure; WBC, white blood cell; INR, international normalized ratio; PTT, partial thromboplastin time; SOFA, sequential organ failure assessment; APSIII, acute physiology score III; OASIS, Oxford acute severity of illness score.

**Table S6. Univariate and multivariate logistic regression analysis of factors influencing AKI.**

| **Variables** | **Univariate analysis** | |  | **Multivariate analysis** | |
| --- | --- | --- | --- | --- | --- |
|  | **OR (95%CI)** | ***P*-value** |  | **OR (95%CI)** | ***P*-value** |
| SOSM |  |  |  |  |  |
| T1 | 0.97 (0.85-1.10) | 0.618 |  | 0.99 (0.86-1.15) | 0.915 |
| T2 | Reference |  |  | Reference |  |
| T3 | 1.69 (1.47-1.93) | <0.001 |  | 1.22 (1.04-1.42) | 0.012 |
| Age | 1.02 (1.01-1.02) | <0.001 |  | 1.01 (1.01-1.02) | <0.001 |
| Gender, Male | 0.92 (0.82-1.04) | 0.176 |  |  |  |
| Race, White | 0.98 (0.87-1.10) | 0.716 |  |  |  |
| BMI | 1.05 (1.04-1.06) | <0.001 |  | 1.07 (1.06-1.08) | <0.001 |
| Heart Rate | 1.01 (1.01-1.01) | 0.009 |  | 0.99 (0.99-0.99) | <0.001 |
| MBP | 0.98 (0.98-0.99) | <0.001 |  |  |  |
| Respiratory Rate | 1.07 (1.05-1.09) | <0.001 |  |  |  |
| Temperature | 1.00 (0.90-1.11) | 0.988 |  |  |  |
| Hemoglobin | 0.99 (0.96-1.02) | 0.445 |  |  |  |
| Platelets | 1.00 (1.00-1.00) | 0.799 |  |  |  |
| WBC | 1.03 (1.02-1.04) | <0.001 |  |  |  |
| Aniongap | 1.09 (1.08-1.11) | <0.001 |  | 1.05 (1.03-1.07) | <0.001 |
| Creatinine | 1.17 (1.12-1.22) | <0.001 |  |  |  |
| INR | 1.27 (1.15-1.40) | <0.001 |  |  |  |
| PTT | 1.01 (1.01-1.01) | <0.001 |  | 1.01 (1.01-1.01) | <0.001 |
| Hypertension | 0.83 (0.74-0.93) | 0.001 |  |  |  |
| Diabetes | 1.15 (1.03-1.28) | 0.014 |  | 0.87 (0.76-0.98) | 0.027 |
| Diabetes with complication | 1.16 (1.01-1.33) | 0.034 |  |  |  |
| Congestive heart failure | 1.78 (1.60-1.99) | <0.001 |  | 1.44 (1.27-1.64) | <0.001 |
| Cerebrovascular disease | 1.40 (1.19-1.65) | <0.001 |  | 1.21 (1.02-1.44) | 0.034 |
| Peripheral vascular disease | 1.34 (1.15-1.56) | <0.001 |  |  |  |
| Chronic pulmonary disease | 1.12 (0.98-1.27) | 0.087 |  |  |  |
| Renal disease | 1.39 (1.23-1.56) | <0.001 |  | 0.85 (0.74-0.99) | 0.037 |
| SOFA | 1.19 (1.17-1.21) | <0.001 |  |  |  |
| APSIII | 1.04 (1.03-1.04) | <0.001 |  | 1.02 (1.01-1.02) | <0.001 |
| OASIS | 1.09 (1.08-1.10) | <0.001 |  | 1.06 (1.05-1.07) | <0.001 |
| Vasoactive | 2.18 (1.95-2.44) | <0.001 |  | 1.58 (1.39-1.80) | <0.001 |
| Antihypertensive | 1.23 (1.04-1.46) | 0.016 |  | 1.43 (1.18-1.73) | <0.001 |
| Antilipemic | 0.93 (0.83-1.06) | 0.277 |  |  |  |
| Antiplatelet | 1.79 (1.42-2.26) | <0.001 |  | 1.48 (1.15-1.91) | 0.003 |
| Insulin | 1.22 (1.10-1.36) | <0.001 |  |  |  |

Abbreviations: AKI, acute kidney injury; OR, odds ratio; CI: confidence interval; SOSM, serum osmolality; BMI, body mass index; MBP, mean blood pressure; WBC, white blood cell; INR, international normalized ratio; PTT, partial thromboplastin time; SOFA, sequential organ failure assessment; APSIII, acute physiology score III; OASIS, Oxford acute severity of illness score.


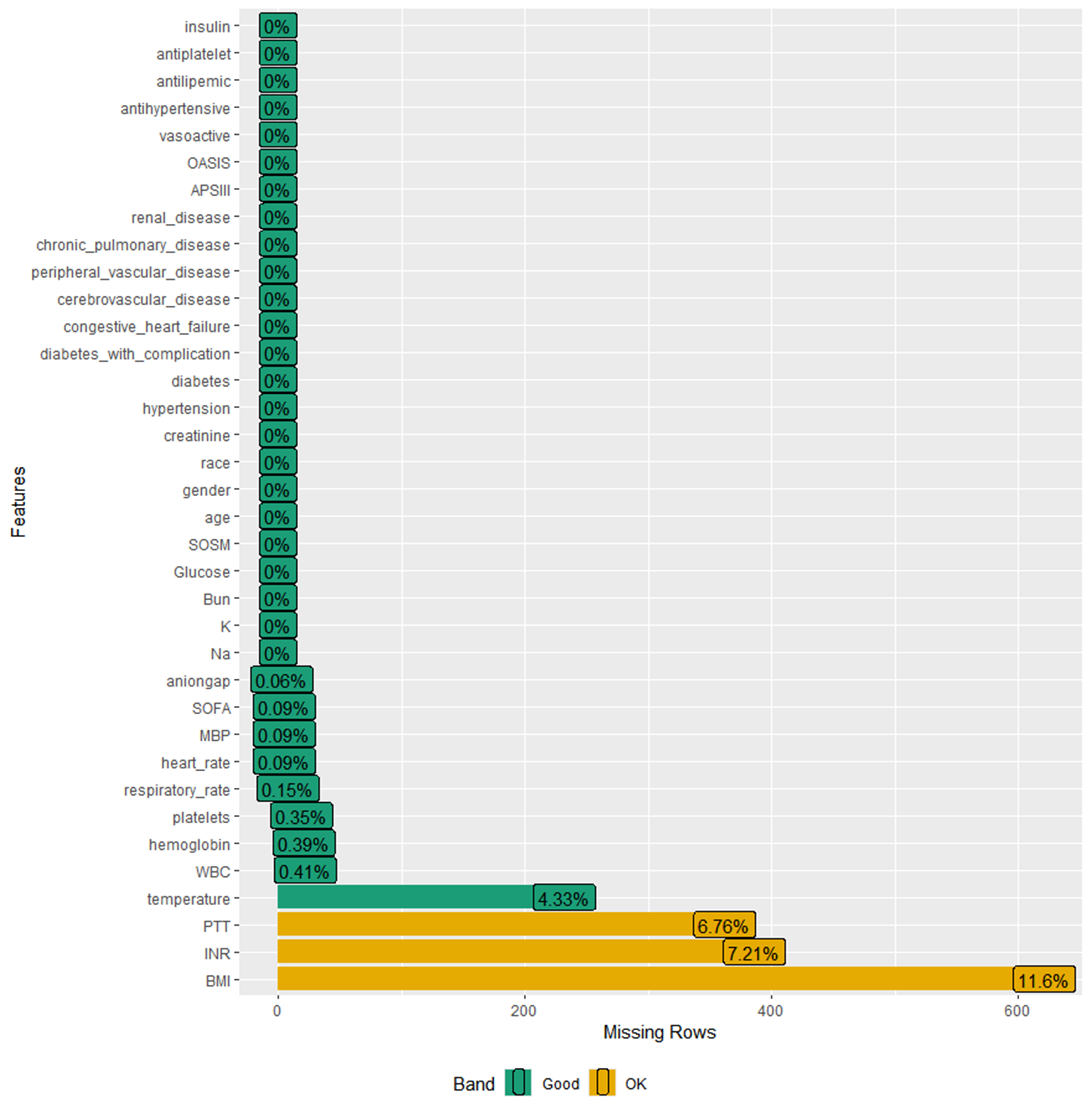


**Figure S1. Percentage of missing data in the variables included in this study.**

Abbreviations: OASIS, Oxford acute severity of illness score; APSIII, acute physiology score III; SOSM, serum osmolality; Bun: blood urea nitrogen; SOFA, sequential organ failure assessment; MBP, mean blood pressure; WBC, white blood cell; PTT, partial thromboplastin time; INR, international normalized ratio; BMI, body mass index.


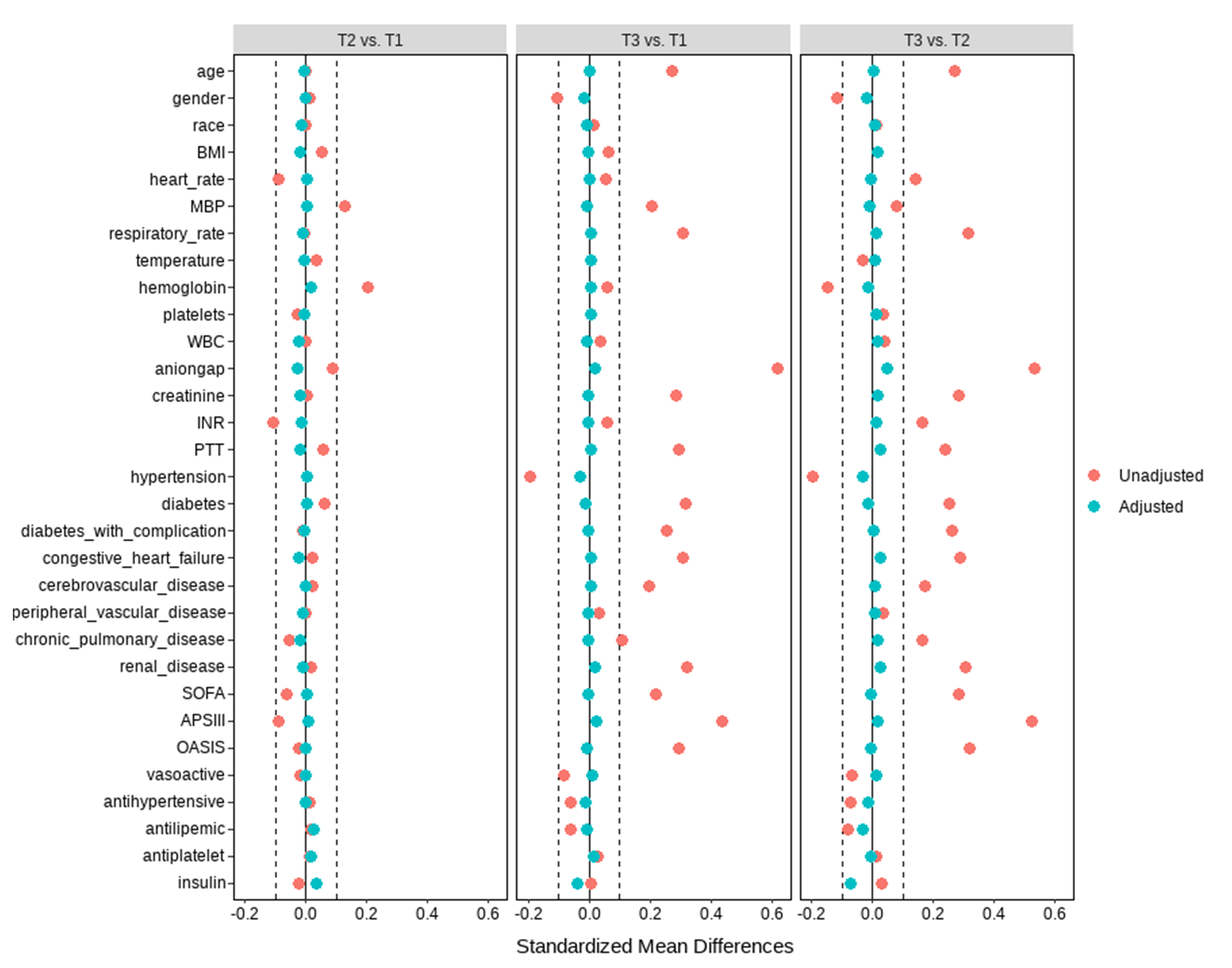


**Figure S2. Love plot of covariates before / after adjustment by IPTW among three groups.**

Abbreviations: IPTW, inverse probability of treatment weighting; BMI, body mass index; MBP, mean blood pressure; WBC, white blood cell; INR, international normalized ratio; PTT, partial thromboplastin time; SOFA, sequential organ failure assessment; APSIII, acute physiology score III; OASIS, Oxford acute severity of illness score.
